# Screening of family members of patients with acute brucellosis in an endemic area of Iran

**Published:** 2013-09

**Authors:** Masoomeh Sofian, Leila Safaeipour, Arezoo Aghakhani, Mohammad Reza Sharif, Mohammad Banifazl, Alireza Sharif, Ali-Asghar Farazi, Ali Eslamifar, Farshideh Didgar, Amitis Ramezani

**Affiliations:** 1 Tuberculosis and Pediatric Infectious Research Center, Arak University of Medical Sciences, Arak, Iran; 2 Arak University of Medical Sciences, Arak, Iran; 3 Clinical Research Dept., Pasteur Institute of Iran, Tehran, Iran; 4 Department of Pediatrics, Kashan University of Medical Sciences, Kashan, Iran; 5 Iranian society for support of patients with infectious disease, Tehran, Iran; 6 Department of Infectious Diseases, Kashan University of Medical Sciences, Kashan, Iran

**Keywords:** Acute Brucellosis, Family member, Screening, Index cases

## Abstract

**Background and Objectives:**

Brucellosis is a zoonotic disease and it's still endemic in Iran. There are some reports regarding brucellosis infection in family members sharing same risk factors and remain unrecognized. However****, few studies on the importance of family screening are available. We aimed to screen household members of index cases with acute brucellosis for detecting additional unrecognized cases in central province of Iran.

**Patients and Methods:**

163 family members of 50 index cases were enrolled in the study. Standard Tube Agglutination Test (STA) and 2-mercaptoethanol (2ME) agglutination were checked in all samples. A case with STA titer ≥ 1:80, 2-mercaptoethanol (2ME) agglutination ≥ 40 and compatible signs and symptoms was considered positive for brucellosis.

**Results:**

15 (9.2%) of family members were seropositive fo**r** Brucella agglutinin and among them, 8 (53.3%) were asymptomatic and 7 (46.7%) were symptomatic. STA titer ranged from 1:80 to 1:640 in seropositive members. 4 of the 15 seropositive cases who identified by screening came from one index case with 6 family members. All symptomatic seropositive cases treated for Brucella infection and recovered without any complications in 6 months follow up.

**Conclusion:**

On the basis of our data, family members of brucellosis patients are at risk of disease acquisition, and screening of household members provides an effective way for early diagnosis and prompt treatment. However cost benefit of screening should be evaluated to reach definite decision for the implementation of the screening as a nationwide program.

## INTRODUCTION

Brucellosis is a zoonotic disease and despite all attempts to reduce it through vaccination of cattle and pasteurization of dairy products, disease still remains an important public health problem in most parts of the world, especially in Asia and the Mediterranean areas (1, 2). The frequency of brucellosis in Iran was estimated from 0.5% to 10.9% in different districts. It's highly endemic in some areas like central province of Iran with five-year incidence rate about 40.5-48/100000 of populations (3).

Human brucellosis is an acute febrile illness and involves multiple organs with a very variable clinical spectrum. The most common manifestations are fever, night sweating, chills, fatigue, malaise, headaches, myalgias and arthralgia (4, 5). Infection can be transmitted to human through direct contact with infected animals or their secretions, consumptions of unpasteurised milk and dairy products and inhalation of aerosols (6, 7). Other rare routes of transmission are vertical (mother to child), through breast feeding, consumption of uncooked meat and sexual contacts (8–10). A few reports have highlighted a high incidence of brucellosis among family members of an index case with brucellosis, due to sharing the same source of infection and similar risk factors such as consumption of unpasteurized dairy products (2, 11–14).

Brucellosis often encounter to a diagnostic challenge due to non-specific and variable clinical presentation and its chronic course (15). Besides the difficulty in the diagnosis, patient may even present late in the course of the disease with a complication in several organs (2, 16). However, early diagnosis and treatment of infected cases can reduce the rate of complication and relapses (13).

Due to the public health implications of brucellosis, this study was carried out to investigate whether active screening of household members of index cases with acute brucellosis can detect additional unrecognized cases in central province of Iran as an endemic area for brucellosis or not.

## PATIENTS AND METHODS

In this prospective epidemiological survey, 163 household members of 50 patients with acute brucellosis (index case) who were attending to health centers were enrolled from March to April 2012 in Arak, Iran. The project was approved by Arak University of Medical Sciences Ethical Committee. Informed consent was provided from all cases. Participants completed a brief questionnaire on demographic, characteristics, clinical and epidemiological data through face to face interview by a single physician.

### Definition

An index case (acute brucellosis) was defined as a patient, who had provided a clinical diagnosis of brucellosis on the basis of the compatible signs and symptoms, Standard Tube Agglutination Test (STA) ≥1:80, and 2-mercaptoethanol (2ME) agglutination ≥ 40.

A family member was defined as a household member who lives in the same house as an index case and sharing similar risk factors. A seropositive family member was defined as a family member with STA ≥ 1:80, and 2ME ≥ 40, with or without brucellosis sign and symptoms.

After diagnosis of index case, family member screening was initiated. We followed up seronegative cases for signs and symptoms of brucellosis at 6 months. Also all symptomatic seropositive cases were treated and followed up for 6 months in order to evaluating response to treatment, relapse, sequel and complication of brucellosis but asymptomatic seropositive subjects which didn't have indication for treatment, didn't cooperate for follow up.

### Statistical analysis

Data were analyzed by the Chi-square test using the SPSS 16.0 data analysis software package; P values <0.05 were considered statistically significant. Data are presented as mean±SD or, when indicated, as an absolute number and percentage.

## RESULTS

A total of 163 family members of 50 index cases were enrolled in the study. The median age of family members was 25 years old. 81 (49.7%) were male and 82 (50.3%) were female. 87.7% of them were habitant in rural area. 58.9% had history of consumption of unpasteurized dairy products. 71.8% of cases kept cattle. 11 (6.7%) of subjects had previous history of brucellosis.

Among family members, 15 (9.2%) were seropositive for *Brucella* agglutinin (titer 1:80 to 1:640). Epidemiological and clinical characteristics of family members were shown in Table 1. Among seropositive members, 8 (53.3%) were asymptomatic and 7 (46.7%) were symptomatic. In symptomatic household members, fever (57%) and night sweating (57%) were the most common manifestation and the other symptoms were headache, arthralgia, anorexia and low back pain each one 14.3%. Five patients had more than one symptom.

**Table 1:** Epidemiological and clinical characteristics of household members of patients with acute brucellosis.

Variable	Household members (n = 163)		P value

	Seropositive (n = 15)	Seronegative (n = 148)		
Age (years)			
≤10	0	27	NS
11–20	1	32	
21–30	6	38	
31–40	2	19	
41–50	3	15	
≥51	3	17	
Sex			
Male	7 (46.7%)	74 (50%)	NS
Female	8 (53.3%)	74 (50%)	
Area of residence			
Urban	2 (13.3%)	18 (12.2%)	NS
Rural	13 (86.7%)	130 (87.8%)	
Consumption of unpasteurized dairy products	8 (53.3%)	88 (59.5%)	NS
Keeping cattle	14 (93.3%)	103 (69.6%)	NS
Type of animals owned			
Sheep	11 (73.3%)	63 (42.6%)	NS
Cow	0 (0%)	14 (9.5%)	
Sheep and cow	3 (20%)	26 (17.6%)	
Previous history of brucellosis	1 (6.7%)	10 (6.8%)	NS

NS: Not significant

STA titer ranged from 1:80 to 1:640 in asymptomatic (4 patients had a titer of 1/80, 3 cases had 1:160 titer, and only 1 case showed a titer of 1:640). In symptomatic family members, STA titer were also from 1:80 to 1:640 (5 cases had 1:80, 1 had 1:320 and 1 had 1:640 titers). 2-ME ranged from 1:40 to 1:320 in both symptomatic and asymptomatic cases.

Four of the 15 seropositive cases identified by screening, came from one index case (with 6 family members) and in the remaining seropositive subjects only one household was infected. All symptomatic seropositive cases treated with *Brucella* medication and recovered without any sequelae, relapse or complication in 6 months follow up but asymptomatic seropositive subjects (which didn't have indication for treatment) didn't participate and cooperate for 6 months follow up.

## DISCUSSION

In this study, we investigated the need for active surveillance of family members of patients with acute brucellosis to enhance detection of unrecognized cases in an endemic area for brucellosis. This survey showed that the seroprevalence of *Brucella* agglutinin in family members was 9.2% and 46.7% of them was symptomatic and infected with brucellosis. This rate was lower than some other results reported earlier from Southeast of Iran, Turkey and Saudi Arabia and higher than Peruvian study. It may be due to difference in predisposing factors such as drinking and eating habits and the degree of exposure.

Two separate studies conducted in Saudi Arabian family members of brucellosis cases showed 19% and 13% of them were seropositive for *Brucella* agglutinin (13, 14). Tabak, et al., (17) from Turkey found the *Brucella* seropositivity rate of 18.2% among family members of index cases with brucellosis, and among seropositive cases, 40% was symptomatic. In an earlier study from Peru, Gotuzzo and colleagues reported that the attack rate of brucellosis among family members of patients with osteoarticular complications was 50.9% after 4 months following diagnosis of the index case (11). A recent study carried out after 2 decades in Peru on household members of acute brucellosis cases showed that 7.3% of family members had serological evidence of brucellosis but 93.3% of them had brucellosis infection (18).

Two separate studies were carried out in North and Southeast of Iran on seroprevalence of brucellosis in family members of index cases (16, 19). Hasanjani Roushan et al., (16) investigated 469 household members and found that 9.6% had two cases of the disease in the same household. In a study in Southeast of Iran by Sharifi-Mood *et al*., (19) 20% of the family members were seropositive for brucellosis and 61% were symptomatic with high titer of *Brucella* agglutinin (>1:640). While in current study in central province of Iran, the prevalence of *Brucella* antibodies was lower than the Southeast (19) but similar to North of Iran (16). However, we acknowledge the lack of control group in our study design as a limitation.

We also found only 1 symptomatic case with *Brucella* agglutinin 1:640 and most cases showed STA titers from 1:80 to 1:160. One of household members was seronegative and asymptomatic in screening time but after 1 week he became seropositive and symptomatic with clinical manifestation of acute brucellosis. Therefore the time of screening (the interval between index case clinical manifestation and seropositivity of new case) is very important in finding new cases in household members of acute brucellosis patients.

Taken together, several studies including our previous report showed that family history and household members have a significant association with *Brucella* seropositivity due to the shared environment and dairy products (2, 20, 21). However it should be considered that genetic factors may affect susceptibility to brucellosis and contribute to a high attack rate in household members exposed to the pathogen (22, 23).

The most benefit of screening the family members is detection of new cases that are unaware of their disease because of mild symptoms such as fever and myalgia which were experienced in other diseases and did not require medical advice. These cases would not have been recognized without screening. But early detection and prompt treatment of the disease can provide rapid recovery and prevent complications or sequels (4, 24, 25). In current study, the prognosis of symptomatic family members after treatment was excellent and all cases recovered without any complication. This result is in accordance with Alsubaie (14) and Tabak et al., (17) investigations. Therefore, the advantages of a screening program are early detection and early treatment which result in fewer complications.

In conclusion, our survey showed that the seroprevalence of brucellosis in household members was 9.2% and active serological screening of family members detected additional symptomatic and asymptomatic seropositive cases. However cost benefit of screening should be considered and evaluated in relation to other public health issues to reach definite conclusion for implementation of screening as a nationwide program.
